# Propofol Use for Sedation in Endoscopic Procedures: Too Many Physicians in the Room?

**DOI:** 10.1093/jcag/gwz021

**Published:** 2019-06-28

**Authors:** Mohammad Yaghoobi

**Affiliations:** Division of Gastroenterology, Michael G. DeGroote School of Medicine, McMaster University and McMaster University Medical Center, Hamilton, Ontario, Canada

In the previous issue of JCAG, Heron et al. published a single-centre retrospective study of close to 5000 patients who underwent endoscopic procedures with moderate/conscious sedation using nonanaesthesiologist-administered propofol sedation, as an adjunct agent. Propofol was administered by gastroenterologists certified in Advanced Cardiovascular Life Support (ACLS). This is also commonly known as balanced propofol sedation (1).

In accordance to other North American professional gastroenterology and endoscopy societies, the Canadian Association of Gastroenterology published a position statement in 2008 highlighting the safety of endoscopist-administered propofol for conscious sedation. Propofol provides quicker onset of action and shorter recovery time than conventional sedation (2). However, this statement recommended formal ACLS certification and further education on the use of propofol when contemplating the practice. The suggested single agent loading dose was 40 to 50 mg followed by smaller bolus loads of 10 to 20 mg. Adding small doses of benzodiazepines and/or opiates will lower the required dose of propofol to achieve moderate rather than deep sedation (3,4). Routine presence of anaesthesiologists for endoscopic procedures was not suggested. One should note that the average dose of 34.5 ± 20.8 mg for propofol used in this study, is considerably less than its standard dose as a single agent for deep sedation. This is very likely due to a synergistic effect when added to a narcotic and a benzodiazepine.

Despite these recommendations, the current most widely used regimen for moderate sedation in Canada is a combination of a narcotic analgesic and a benzodiazepine, in the absence of a contraindication. Propofol, occasionally in combination with an analgesic or a benzodiazepine, is the most commonly used agent for deep sedation in Canada. It is most commonly administered by an anaesthesiologist, who can also provide airway support.

In this study, the safety profile of propofol, as an added agent to standard dual moderate sedation regimen, is established in a relatively large population. The absence of any serious complications, including death or need for mechanical ventilation, is reassuring given the large sample size of the study and the variety of included procedures. In a larger previous multicenter retrospective study of more than 36,000 endoscopies, episodes of apnea or other airway compromise requiring assisted ventilation occurred in less than 0.2% and there was no endotracheal intubation, permanent injury or death (5). Moreover, two randomized controlled trials compared endoscopist-administered mono-propofol sedation with different combinations of moderate sedations regimens (6,7). Patients receiving propofol showed significantly shorter recovery time with no significant differences in major complications, physician satisfaction, or patient-reported pain or discomfort. Patient satisfaction across all controlled trials was also better with mono-propofol sedation.

Adding propofol may also enhance cost-effectiveness and patient safety, although this will require further studies. While the safety profile of propofol as single or adjunct agent has been demonstrated in several studies, endoscopists should still personalize their care and keep in mind the importance of patient selection (including ASA classification), informed consent, availability of trained personnel, physician comfort level and required training before deciding which agent(s) and dose to use. Trained individuals should be present during the procedure to monitor airways and physiologic parameters. Overall, nonanaesthesiologist-administered propofol sedation might improve patient comfort, procedural efficiency, and/or successful procedure completion. However, local regulations regarding administration of propofol should be sought and followed.

Last but not least, caution should be exercised when an endoscopic procedure is performed in an out-of-hospital facility, noting that the study by Heron et al. was conducted in a hospital setting. Propofol as single deep sedation agent, administered by an anaesthesiologist, should be reserved for selected patients who may not be appropriate candidates for nonanesthesiologist-administered sedation.

## References

[1] Early DS, Lightdale JR, Vargo JJ II, et al; ASGE Standards of Practice Committee. Guidelines for sedation and anesthesia in GI endoscopy. Gastrointest Endosc 2018;87(2):327–37. 29306520 10.1016/j.gie.2017.07.018

[2] Byrne MF, Chiba N, Singh H, et al; Clinical Affairs Committee of the Canadian Association of Gastroenterology. Propofol use for sedation during endoscopy in adults: A Canadian Association of Gastroenterology position statement. Can J Gastroenterol 2008;22(5):457–9. 18478130 10.1155/2008/268320 PMC2660799

[3] Cohen LB, Hightower CD, Wood DA, et al Moderate level sedation during endoscopy: A prospective study using low-dose propofol, meperidine/fentanyl, and midazolam. Gastrointest Endosc 2004;59(7):795–803. 15173791 10.1016/s0016-5107(04)00349-9

[4] VanNatta ME, Rex DK Propofol alone titrated to deep sedation versus propofol in combination with opioids and/or benzodiazepines and titrated to moderate sedation for colonoscopy. Am J Gastroenterol 2006;101(10):2209–17. 17032185 10.1111/j.1572-0241.2006.00760.x

[5] Rex DK, Heuss LT, Walker JA, et al Trained registered nurses/endoscopy teams can administer propofol safely for endoscopy. Gastroenterology 2005;129(5):1384–91. 16285939 10.1053/j.gastro.2005.08.014

[6] Ulmer BJ, Hansen JJ, Overley CA, et al Propofol versus midazolam/fentanyl for outpatient colonoscopy: Administration by nurses supervised by endoscopists. Clin Gastroenterol Hepatol 2003;1(6):425–32. 15017641 10.1016/s1542-3565(03)00226-x

[7] Sipe BW, Rex DK, Latinovich D, et al Propofol versus midazolam/meperidine for outpatient colonoscopy: Administration by nurses supervised by endoscopists. Gastrointest Endosc 2002;55(7):815–25. 12024134 10.1067/mge.2002.124636

